# The association between academic major identity and career decision-making difficulty among Chinese college students: a sequential indirect association model of psychological capital and career adaptability

**DOI:** 10.3389/fpsyg.2026.1869688

**Published:** 2026-06-17

**Authors:** Rui Mou, Xiaoxiao Han, Zhenzhen Jia

**Affiliations:** 1 Department of External Cooperation, Sichuan Technology and Business University, Meishan, China; 2 School of Health Management, Weifang Nursing Vocational College, Weifang, China

**Keywords:** academic major identity, career adaptability, career decision-making difficulty, Chinese college students, psychological capital

## Abstract

Academic major identity, referring to students’ cognitive, affective, behavioral, and fit-based identification with their field of study, may be an important correlate of career decision-making difficulty. Career adaptability refers to psychosocial resources that individuals use to manage career-related tasks, transitions, and uncertainty. Drawing on career construction theory, this cross-sectional study examined a theoretically informed model of associations among academic major identity, psychological capital, career adaptability, and career decision-making difficulty among Chinese college students. Data were collected through an anonymous online survey using Questionnaire Star. Participants were 2,255 first-, second-, and third-year undergraduate students recruited from 20 universities in Sichuan Province, China. Established instruments were used to measure academic major identity, psychological capital, career adaptability, and career decision-making difficulty. Structural equation modeling and bias-corrected bootstrap procedures were used to analyze the data. The results showed that academic major identity was negatively associated with career decision-making difficulty and positively associated with psychological capital and career adaptability. Psychological capital and career adaptability were both negatively associated with career decision-making difficulty, and psychological capital was positively associated with career adaptability. Bootstrap analyses further indicated significant indirect associations between academic major identity and career decision-making difficulty through psychological capital, through career adaptability, and through the sequential pathway of psychological capital and career adaptability. These findings should be interpreted as associations rather than causal effects because of the cross-sectional design. Overall, the study contributes to the literature by integrating major-related identification, general positive psychological resources, and career-specific adaptive resources within a single analytical framework. The Chinese higher education context, characterized by major placement, family expectations, and increasingly diversified post-graduation pathways, makes this integrated model particularly relevant for understanding career decision-making difficulty among undergraduates.

## 1 Introduction

With the expansion of higher education and the increasing diversification of post-graduation pathways in China, career development has become a major developmental task for college students (Ding et al., 2021). For Chinese college students, career-related choices extend far beyond the question of whether to seek employment after graduation (Yang et al., 2024). They often involve multiple alternatives, such as postgraduate study, civil service examinations, public institution recruitment, flexible employment, and cross-disciplinary career development (Shi, 2023; Song et al., 2024). In this context, students are expected to integrate information about their interests, abilities, academic backgrounds, family expectations, and labor market opportunities, making career decision-making a complex and demanding process. As a result, career decision-making difficulty has become an important topic in research on college students’ career development (Xu and Bhang, 2019; Xu, 2024). Career decision-making difficulty is typically reflected in problems such as insufficient occupational information, unclear self-understanding, vague career planning, and uncertain career goals (Xu, 2024; Udayar et al., 2020). These difficulties are not only relevant to students’ current career exploration but are also closely related to their transition from university to broader social and occupational contexts (Chen W. et al., 2025; Arbona et al., 2021). Accordingly, examining factors associated with career decision-making difficulty among Chinese college students is of clear theoretical and practical significance.

Among the factors relevant to college students’ career development, academic major identity deserves particular attention. Academic major identity refers to students’ overall identification with their academic major in terms of cognition, affect, behavioral engagement, and perceived fit (Wang H. et al., 2023). In the Chinese context, students’ choice of academic major is not always based solely on personal interest. It may also be shaped by the college entrance examination system, admission adjustment, parental expectations, and perceived employment prospects (Zhu et al., 2024). Consequently, after entering university, many students continue to evaluate, adapt to, and make sense of their academic majors. A higher level of academic major identity generally reflects clearer recognition of the value of one’s major, stronger emotional acceptance, greater willingness to engage in major-related activities, and a stronger sense of fit between the self and the major (Lehman and Nauta, 2022; You, 2018). From a career development perspective, students with higher academic major identity may be more likely to connect their academic training with future career directions and may therefore report fewer difficulties in occupational information integration, self-exploration, and goal clarification (Wessel et al., 2008; Gerçek and Elmas-Atay, 2022). By contrast, when students show a weaker sense of identification with their academic major, their career development process may be characterized by greater hesitation, instability, and uncertainty (McLean et al., 2022). Therefore, academic major identity may be closely associated with career decision-making difficulty.

In addition to academic major identity, psychological capital may serve as an important positive psychological resource in understanding college students’ career development (Youssef-Morgan, 2024). Psychological capital comprises self-efficacy, hope, resilience, and optimism, and reflects an individual’s positive psychological state when dealing with developmental tasks and future challenges (Zhou A. et al., 2024; Xu et al., 2023a). For college students, a stronger sense of identification with one’s academic major may be associated with more positive academic experiences and a clearer sense of developmental direction, which may in turn relate to higher psychological capital (Ayala Calvo and Manzano García, 2020; Belle et al., 2021). At the same time, students with higher psychological capital may be more likely to maintain confidence, persistence, and positive expectations in the face of career exploration and career uncertainty, and may therefore report lower levels of career decision-making difficulty (Hassan et al., 2023). In this sense, psychological capital may help explain why academic major identity is associated with career decision-making difficulty.

Career adaptability is another important construct for understanding individual differences in career development (Rudolph et al., 2017). According to career construction theory, career adaptability refers to the psychosocial resources individuals draw upon to cope with current and anticipated career-related tasks, transitions, and uncertainties. It includes four dimensions: concern, control, curiosity, and confidence (Savickas, 2005). For Chinese college students, the transition from academic learning to future occupational development is an ongoing process of preparation, exploration, and adjustment (Pan et al., 2018; Xu et al., 2023b). In this process, career adaptability is particularly relevant. Students with higher academic major identity may be more likely to maintain future-oriented concern, engage more actively in career-related exploration, and show greater control and confidence in career planning (Myint and Robnett, 2024; Wang et al., 2025). These characteristics suggest a possible positive association between academic major identity and career adaptability. In turn, students with higher career adaptability may be better able to deal with uncertainty, gather and integrate career-related information, and clarify their vocational goals, and may therefore experience lower career decision-making difficulty (Leung et al., 2021; Santos et al., 2018). Thus, career adaptability may also help explain the association between academic major identity and career decision-making difficulty.

The relationship between psychological capital and career adaptability is also theoretically meaningful. Psychological capital represents a relatively general set of positive psychological resources, whereas career adaptability reflects more domain-specific resources for managing career development tasks (Zhou W. et al., 2024; Xu M. et al., 2023). For college students, stronger self-efficacy, hope, resilience, and optimism may be associated with greater concern for the future, stronger self-regulation in career planning, broader curiosity about occupational possibilities, and greater confidence in dealing with career-related tasks (Zyberaj et al., 2022). From this perspective, psychological capital may provide a broader psychological foundation that is statistically linked to career-specific adaptive resources. Accordingly, psychological capital may be associated not only with career decision-making difficulty directly but also indirectly through career adaptability (Kvasková et al., 2022). Based on this logic, academic major identity, psychological capital, career adaptability, and career decision-making difficulty may form a theoretically ordered pattern of associations in which stronger major-related identification is linked to higher positive psychological resources, higher career-specific adaptive resources, and lower career decision-making difficulty (Jiang et al., 2022; Gerçek and Elmas-Atay, 2022).

From the perspective of career construction theory, this integrated pattern can be understood as a process of meaning construction and adaptive resource development. Career construction theory emphasizes that individuals respond to career-related tasks and transitions by constructing meaning and drawing on psychosocial resources (Savickas, 2005). In the university context, academic major identity may represent an important form of major-related meaning construction because it reflects how students understand their academic experiences, evaluate the value of their major, and connect their field of study with possible future careers. Psychological capital may represent a broader layer of positive psychological resources that supports students in approaching developmental uncertainty with efficacy, hope, resilience, and optimism. Career adaptability, in contrast, may represent a more career-specific layer of adaptive resources that enables students to manage career-related tasks, transitions, and uncertainty. Therefore, the association between academic major identity and career decision-making difficulty may be better understood by considering both general positive psychological resources and career-specific adaptive resources within a single framework.

This framework is especially relevant in the Chinese higher education context. In China, academic major experiences are closely connected with institutional placement, family expectations, and perceived employment prospects. Students’ identification with their major may therefore reflect not only personal interest but also their ongoing adjustment to a major that may have been shaped by examination performance, admission policies, parental expectations, and labor market considerations (Zhu et al., 2024; Yang et al., 2024). At the same time, Chinese college students face increasingly diversified post-graduation pathways, including employment, postgraduate study, civil service examinations, public institution recruitment, flexible employment, and cross-disciplinary development. These contextual features make it important to examine how major-related identification, positive psychological resources, and career-specific adaptive resources are jointly associated with career decision-making difficulty among Chinese undergraduates.

Although previous studies have examined the links among academic experiences, positive psychological resources, career adaptability, and various career-related outcomes, several gaps remain (Xu et al., 2025; Adil et al., 2020). First, compared with constructs such as learning engagement, career maturity, and career decision-making self-efficacy, career decision-making difficulty has received relatively less attention in integrated models involving both academic and psychological factors. Second, research simultaneously considering academic major identity, psychological capital, and career adaptability in relation to career decision-making difficulty remains limited. Third, in the Chinese educational context, students’ academic major experiences are closely intertwined with their future career development, making it important to examine these associations within a Chinese college student sample (Yang et al., 2024; Xiong et al., 2026; Zhou A. et al., 2024). Therefore, the present study focused on Chinese college students and examined the associations among academic major identity, psychological capital, career adaptability, and career decision-making difficulty.

**Figure 1:**
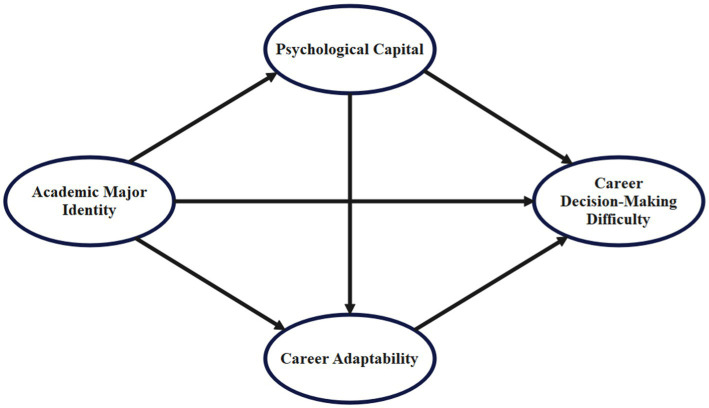
Proposed conceptual model of the associations among academic major identity, psychological capital, career adaptability, and career decision-making difficulty. Arrows represent hypothesized statistical associations rather than causal effects.

As shown in Figure 1, the present study proposed a conceptual model in which academic major identity is associated with career decision-making difficulty both directly and indirectly through psychological capital and career adaptability. The directional paths in the model represent theoretically specified statistical associations rather than causal effects. Specifically, the following hypotheses were proposed:

*H1*: Academic major identity is negatively associated with career decision-making difficulty among Chinese college students.

*H2*: Academic major identity is indirectly associated with career decision-making difficulty through psychological capital.

*H3*: Academic major identity is indirectly associated with career decision-making difficulty through career adaptability.

*H4*: Academic major identity is indirectly associated with career decision-making difficulty through the sequential pathway of psychological capital and career adaptability.

## 2 Methods

### 2.1 Participants and procedure

This study employed a cross-sectional, anonymous online survey design. Data were collected from January 15, 2026 to February 15, 2026, using Questionnaire Star (Wenjuanxing), a widely used online survey platform in China. The target population comprised full-time undergraduate students in Years 1 to 3 enrolled at universities in Sichuan Province, China. A convenience sampling approach was used. With the assistance of faculty members, counselors, and student coordinators at participating universities, the survey link was disseminated to eligible students through routine student communication channels, such as class-based online groups, departmental notices, and messages forwarded through university or college-level student communication networks. Participation was entirely voluntary and anonymous. No monetary or course-credit incentives were provided. Students could access the questionnaire only after reading the online informed consent form and indicating their consent. Because the survey link was distributed through routine student communication channels rather than through a centralized sampling frame, the exact number of students who received the invitation could not be determined; therefore, a population-level response rate could not be calculated.

The survey involved students from 20 universities in Sichuan Province, namely Sichuan Technology and Business University, Sichuan University, Southwest Jiaotong University, University of Electronic Science and Technology of China, Southwest Petroleum University, Chengdu University of Technology, Southwest University of Science and Technology, Chengdu University of Information Technology, Sichuan University of Science and Engineering, Xihua University, Civil Aviation Flight University of China, Sichuan Agricultural University, Sichuan Normal University, China West Normal University, Mianyang Normal University, Southwestern University of Finance and Economics, Chengdu Sport University, Chengdu University, Chengdu Technological University, and Sichuan Tourism University. Given the use of convenience sampling and voluntary participation, the final sample should be understood as a regional convenience sample rather than a probability sample of all Chinese college students.

Prior to participation, all respondents were directed to an online informed consent page that explained the purpose of the study, the voluntary nature of participation, the anonymous handling of responses, and the use of the data for academic research only. Participants could proceed to the questionnaire only after indicating their consent online, and they could discontinue participation before submission. Thus, all participants provided informed consent before completing the survey. The study protocol was reviewed and approved by the Ethics Committee of Sichuan Technology and Business University (Approval No. SCGSDWJL2026010502). In the final valid sample, 86 participants were 17 years old and therefore legally minors. For these participants, the ethics committee approved a waiver of parental/guardian consent because the survey posed minimal risk, was fully anonymous, collected no personally identifying information (e.g., names, student IDs, phone numbers, or IP-linked identity records), and focused on non-sensitive educational and career-related perceptions rather than clinically sensitive or high-risk content. Under the approved protocol, these participants were allowed to provide their own online informed consent before participation. All methods were performed in accordance with the relevant ethical guidelines and regulations.

Several design features were implemented to improve data quality and reduce the likelihood of common method bias at the procedural stage. First, participation was anonymous and voluntary, which helped reduce social desirability concerns and evaluation apprehension. Second, respondents were explicitly informed that there were no right or wrong answers and were encouraged to answer as honestly as possible according to their actual experiences. Third, all measures were administered in a standardized online format using established scales and neutral wording. Fourth, the questionnaire platform was configured so that all items were mandatory, thereby preventing item-level missing data. Each IP address was allowed to submit the questionnaire only once, which reduced the possibility of duplicate submissions. In addition, demographic items and the substantive study scales were presented in clearly separated sections with standardized instructions to reduce respondents’ tendency to infer the expected pattern of associations among variables.

A total of 2,571 questionnaires were returned. Because all items were set as mandatory, there were no questionnaires with missing item responses. The returned questionnaires were then screened for response quality before analysis. Questionnaires were excluded if they met one or more of the following criteria: (a) an unrealistically short completion time, defined as less than 5 min; (b) obvious careless or nonserious responding, such as long-string responses, invariant responding, or highly regular response patterns across scales; (c) logically inconsistent demographic information; and/or (d) other evidence of invalid responding identified during data screening. Long-string or invariant responses referred to response patterns in which the same response option was repeatedly selected across a large block of items or across an entire scale, whereas highly regular response patterns referred to mechanical or patterned responses that were unlikely to reflect meaningful item-level evaluation. After data cleaning, 316 questionnaires were removed, yielding 2,255 valid questionnaires, corresponding to a valid-response rate of 87.71% among the returned questionnaires. This valid-response rate refers only to the proportion of usable questionnaires among returned questionnaires, not to a population-level response rate among all invited students.

As shown in Table 1, the final sample included 1,104 males (48.96%) and 1,151 females (51.04%), with ages ranging from 17 to 23 years (M = 19.42, SD = 1.01). In terms of grade level, 796 participants were freshmen (35.30%), 719 were sophomores (31.88%), and 740 were juniors (32.82%). Students came from six broad academic disciplines: Humanities and Social Sciences (17.96%), Science and Engineering (16.14%), Economics and Management (16.45%), Education (16.94%), Medicine (15.92%), and Arts and Sports (16.59%). In addition, 65.54% of the participants were from urban areas and 24.08% reported having internship experience.

**Table 1:** Demographic characteristics of participants (*N* = 2,255).

Characteristic	Category	*n*	%
Gender	Male	1,104	48.96
	Female	1,151	51.04
Age (years)	Mean ± SD	19.42 ± 1.01	
	Range	17–23	
Grade	Freshman	796	35.30
	Sophomore	719	31.88
	Junior	740	32.82
Academic discipline	Humanities and Social Sciences	405	17.96
	Science and Engineering	364	16.14
	Economics and Management	371	16.45
	Education	382	16.94
	Medicine	359	15.92
	Arts and Sports	374	16.59
Place of origin	Urban	1,478	65.54
	Rural	777	34.46
Internship experience	Yes	543	24.08
	No	1712	75.92

Age is presented as mean ± standard deviation; categorical variables are presented as *n* (%).

### 2.2 Measures

All instruments used in the present study were established scales that have been used in Chinese student samples. To ensure consistency in online administration, all measures were presented using a unified 5-point Likert-type response format. This approach was adopted to reduce cognitive switching and response burden when participants completed multiple scales in a single online questionnaire. Because adapting response formats may influence comparability with studies using the original response scales, the reliability, convergent validity, and discriminant validity of all constructs were re-examined in the present sample. Detailed measurement model results for the first-order dimensions and higher-order constructs are reported in Supplementary Table S1.

Academic major identity was assessed using the College Students’ Major Identity Scale developed by Qin (2009). The scale consists of 23 items across four dimensions: cognitive identity (5 items), affective identity (8 items), behavioral identity (6 items), and fit (4 items). The scale is designed to capture students’ overall identification with their academic major in terms of cognitive recognition, emotional acceptance, behavioral engagement, and perceived fit between the self and the major. In the present study, all items were rated on a 5-point scale ranging from 1 = strongly disagree to 5 = strongly agree. A sample item is: “I am willing to work in a job related to my major.” Item scores were averaged to form dimension scores and an overall academic major identity score, with higher scores indicating a stronger level of academic major identity. The Chinese version of this scale has shown satisfactory reliability and validity in previous studies involving Chinese college students (Bai et al., 2025).

Career decision-making difficulty was measured using the short-form Chinese version of the Career Decision-Making Difficulties Questionnaire (CDDQ) revised by Du and Long (2006), based on the original measure developed by Gati et al. (1996). The short form contains 16 items covering four dimensions: difficulties in career information exploration (5 items), difficulties in career self-exploration (4 items), difficulties in career planning exploration (3 items), and difficulties in career goal exploration (4 items). This instrument assesses the extent to which individuals experience difficulty in gathering career-related information, understanding themselves in relation to career choice, planning their future careers, and clarifying career goals. In the present study, all items were rated on a 5-point scale ranging from 1 = strongly disagree to 5 = strongly agree. A sample item is: “I have a clear career development plan.” In the original short-form scoring, higher total scores indicate lower levels of career decision-making difficulty. To maintain conceptual consistency with the construct label used in the present study, each item was reverse-coded before computing dimension and total scores using the transformation 6 − original score. Thus, higher scores in the present study indicated greater career decision-making difficulty. The Chinese short-form version has demonstrated acceptable psychometric properties in prior studies with Chinese college students (Yang et al., 2020; Zhao and Li, 2013; Tien, 2005).

Psychological capital was assessed using the Chinese version of the Psychological Capital Questionnaire (PCQ-24) developed by Wen and colleagues (Wen et al., 2009). The scale includes 24 items and comprises four dimensions: self-efficacy (6 items), hope (6 items), resilience (6 items), and optimism (6 items). It is intended to measure individuals’ positive psychological resources, including confidence in handling challenging tasks, perseverance toward goals, the capacity to recover from setbacks, and positive expectations regarding future outcomes. In the present study, all items were rated on a 5-point scale ranging from 1 = strongly disagree to 5 = strongly agree. A sample item is: “Every problem has many solutions.” After any reverse-worded items in the administered Chinese version were reverse-scored, item scores were averaged to form dimension scores and an overall psychological capital score. Higher scores indicated higher levels of psychological capital. Previous research has supported the reliability and validity of the Chinese version of the PCQ-24 in university student samples (Zhang et al., 2010; Chen Y. et al., 2025; Wang W. et al., 2023).

Career adaptability was measured using the Career Adapt-Abilities Scale (CAAS) developed by Savickas and Porfeli (2012). The scale contains 24 items across four dimensions: concern (6 items), control (6 items), curiosity (6 items), and confidence (6 items). This instrument assesses the psychosocial resources individuals use to cope with current and anticipated career-related tasks, transitions, and uncertainties. In the present study, all items were rated on a 5-point scale ranging from 1 = strongly disagree to 5 = strongly agree. A sample item is: “I am willing to overcome difficulties.” Item scores were averaged to produce dimension scores and an overall career adaptability score, with higher scores indicating stronger career adaptability. The Chinese version of the CAAS has been shown to have good psychometric properties in Chinese samples (Hou et al., 2012; Yu et al., 2020).

### 2.3 Data analysis

Data analysis was conducted using SPSS 27.0 and AMOS 26.0. Because all questionnaire items were set as mandatory in the survey platform, the final dataset contained no item-level missing values. Before the main analyses, the data were screened for invalid responses in accordance with the criteria described in Section 2.1. Descriptive statistics, including means, standard deviations, and Cronbach’s alpha coefficients, were then computed for all study variables. The score for career decision-making difficulty was prepared as described in Section 2.2, so that higher scores indicated greater difficulty and were therefore consistent with the construct label used in the present study.

Several procedures were used to evaluate the measurement properties of the study variables. First, internal consistency was assessed using Cronbach’s alpha (*α*) and composite reliability (CR), with values of 0.70 or above considered acceptable (Nunnally and Bernstein, 1994). Convergent validity was examined using standardized factor loadings and average variance extracted (AVE). Standardized loadings of 0.50 or above were considered acceptable, and AVE values of 0.50 or above indicated adequate convergent validity (Fornell and Larcker, 1981). Discriminant validity was examined using two complementary approaches. Under the Fornell–Larcker criterion, the square root of the AVE for each construct should exceed its correlations with other constructs (Fornell and Larcker, 1981). In addition, the heterotrait–monotrait ratio (HTMT) was calculated, with values below 0.85 indicating relatively stringent evidence of discriminant validity and values below 0.90 indicating acceptable discriminant validity (Henseler et al., 2015).

Given that all substantive variables were collected through self-report questionnaires in a single online session, common method variance was evaluated using multiple diagnostic procedures. Harman’s single-factor test was used only as an initial diagnostic rather than as a standalone test of common method variance, because this approach has recognized limitations (Podsakoff et al., 2003). For Harman’s test, the proportion of variance explained by the first unrotated factor was examined descriptively. In addition, confirmatory factor analytic model comparisons were conducted to provide a more stringent assessment. A series of alternative measurement models were compared, including one-factor, two-factor, three-factor, four-factor, and unmeasured latent method construct (ULMC) models. In the ULMC model, a latent common method factor was added in addition to the four substantive factors. If the hypothesized four-factor model showed a clearly better fit than the more constrained alternative models, and if the inclusion of the common latent method factor did not substantially improve model fit, common method variance was considered unlikely to be the sole explanation for the observed associations. However, because the study relied on self-report data collected at one time point, common method variance could not be completely ruled out.

Pearson correlation analyses were conducted to examine the bivariate associations among academic major identity, psychological capital, career adaptability, and career decision-making difficulty. Structural equation modeling (SEM) was then used to test the hypothesized pattern of direct and indirect associations among the four constructs. Model fit was evaluated using multiple fit indices, including the chi-square statistic (χ^2^), chi-square to degrees of freedom ratio (χ^2^/df), comparative fit index (CFI), Tucker–Lewis index (TLI), root mean square error of approximation (RMSEA), and standardized root mean square residual (SRMR). Because the chi-square statistic is sensitive to sample size, model evaluation was based primarily on approximate fit indices rather than on χ^2^ alone (Kline, 2016). Following commonly used guidelines, CFI and TLI values of 0.90 or above were considered indicative of acceptable fit, with values of 0.95 or above indicating good fit; RMSEA and SRMR values of 0.08 or below indicated acceptable fit, with values of 0.06 or below indicating good fit (Hu and Bentler, 1999; Kline, 2016). The χ^2^/df value was reported as an auxiliary index, with values below 5.00 considered acceptable.

To examine the indirect associations, bias-corrected bootstrap analyses with 2,000 resamples were conducted. The significance of the direct, specific indirect, and sequential indirect associations was determined based on the 95% bias-corrected confidence intervals (CIs). An association was considered statistically significant when the corresponding confidence interval did not include zero. For the structural model, standardized coefficients (*β*), standard errors (SE), critical ratios (C.R.), and *p* values were reported. For the bootstrap analyses, bias-corrected 95% confidence intervals were additionally reported. Unless otherwise specified, statistical significance was defined as *p* < 0.05 (two-tailed).

## 3 Results

### 3.1 Common method bias

Given that all variables were collected using self-report measures at a single time point, common method variance was examined using both Harman’s single-factor test and confirmatory factor analytic approaches. First, an exploratory factor analysis was conducted using Harman’s single-factor test as an initial diagnostic. The results indicated that 16 factors with eigenvalues greater than 1 were extracted, accounting for 65.37% of the total variance. The first unrotated factor explained 25.81% of the variance.

Second, a series of alternative measurement models were compared (see Table 2). The hypothesized four-factor model, including academic major identity, psychological capital, career adaptability, and career decision-making difficulty as four distinct constructs, demonstrated a substantially better fit to the data (χ^2^ = 369.23, df = 98, χ^2^/df = 3.77, CFI = 0.980, TLI = 0.975, RMSEA = 0.035, SRMR = 0.031) than the one-factor, two-factor, and three-factor models. Furthermore, the unmeasured latent method construct (ULMC) model, which included a latent common method factor in addition to the four substantive factors, showed a very similar fit to the four-factor model (χ^2^ = 360.29, df = 97, χ^2^/df = 3.71, CFI = 0.980, TLI = 0.976, RMSEA = 0.035, SRMR = 0.030). The negligible improvement in model fit suggests that the inclusion of a common method factor did not substantially enhance model performance. Taken together, these results suggest that common method variance was unlikely to be the sole explanation for the observed associations among the study variables. Nevertheless, because all variables were measured through self-report questionnaires in a single online session, common method variance could not be completely ruled out.

**Table 2:** Comparison of alternative measurement models for common method bias.

Model	Model specification	χ^2^	df	χ^2^/df	CFI	TLI	RMSEA	SRMR
Model 1	One-factor model: all indicators loaded on a single latent factor	3033.16	104	29.17	0.781	0.747	0.112	0.080
Model 2	Two-factor model: AMI + PsyCap; CA + CDD	2774.16	103	26.93	0.800	0.767	0.107	0.077
Model 3A	Three-factor model A: AMI + PsyCap; CA; CDD	2088.12	101	20.67	0.851	0.823	0.093	0.069
Model 3B	Three-factor model B: AMI + CA; PsyCap; CDD	1464.43	101	14.50	0.898	0.879	0.077	0.057
Model 3C	Three-factor model C: AMI; PsyCap + CA; CDD	1029.10	101	10.19	0.931	0.917	0.064	0.047
Model 3D	Three-factor model D: AMI; PsyCap; CA + CDD	1075.16	101	10.65	0.927	0.913	0.065	0.048
Model 4	Four-factor model: AMI; PsyCap; CA; CDD	369.23	98	3.77	0.980	0.975	0.035	0.031
Model 5	ULMC model: four substantive factors plus one latent common method factor	360.29	97	3.71	0.980	0.976	0.035	0.030

AMI = academic major identity; PsyCap = psychological capital; CA = career adaptability; CDD = career decision-making difficulty. ULMC = unmeasured latent method construct. In the ULMC model, each indicator loaded on its theoretical substantive factor and simultaneously on a latent common method factor. χ^2^/df = chi-square to degrees of freedom ratio; CFI = Comparative Fit Index; TLI = Tucker–Lewis Index; SRMR = Standardized Root Mean Square Residual; RMSEA = Root Mean Square Error of Approximation.

### 3.2 Descriptive statistics and measurement properties

Descriptive statistics and measurement properties for the study variables are presented in Table 3. The reliability indices indicated satisfactory internal consistency for all four higher-order constructs. Cronbach’s *α* coefficients ranged from 0.898 to 0.940, suggesting good reliability. In addition, the standardized factor loadings for the higher-order constructs ranged from 0.723 to 0.778, composite reliability (CR) values ranged from 0.836 to 0.849, and average variance extracted (AVE) values ranged from 0.560 to 0.584. These results support the adequacy of the measurement properties of the four focal higher-order constructs.

**Table 3:** Descriptive statistics and measurement properties of the four higher-order constructs.

**Variable**	**Mean**	**SD**	**α**	**Std. loading range**	**CR**	**AVE**
AMI	3.636	0.706	0.929	0.723–0.768	0.836	0.561
PsyCap	3.567	0.736	0.940	0.739–0.778	0.849	0.584
CA	3.483	0.724	0.937	0.730–0.763	0.836	0.560
CDD	2.442	0.708	0.898	0.736–0.764	0.836	0.561

AMI = academic major identity; PsyCap = psychological capital; CA = career adaptability; CDD = career decision-making difficulty. AMI contained 23 items, PsyCap contained 24 items, CA contained 24 items, and CDD contained 16 items. SD = standard deviation; α = Cronbach’s alpha; CR = composite reliability; AVE = average variance extracted.

Detailed measurement results for the first-order dimensions are presented in Supplementary Table S1. Across the first-order dimensions, Cronbach’s α coefficients ranged from 0.783 to 0.906, CR values ranged from 0.784 to 0.907, and AVE values ranged from 0.510 to 0.620. Although several dimensions of career decision-making difficulty, particularly difficulties in career planning exploration, showed comparatively lower reliability than the corresponding higher-order construct, all reliability and convergent validity indices met commonly accepted thresholds. Therefore, the subsequent structural model was interpreted primarily at the higher-order construct level rather than at the dimension-specific level.

### 3.3 Correlations and discriminant validity

The correlations and Fornell–Larcker results are presented in Table 4. Academic major identity was positively correlated with psychological capital (*r* = 0.358, *p* < 0.001) and career adaptability (*r* = 0.490, *p* < 0.001), and negatively correlated with career decision-making difficulty (*r* = −0.475, *p* < 0.001). Psychological capital was positively correlated with career adaptability (*r* = 0.608, *p* < 0.001) and negatively correlated with career decision-making difficulty (*r* = −0.482, *p* < 0.001). Career adaptability was also negatively correlated with career decision-making difficulty (*r* = −0.536, *p* < 0.001). Overall, the zero-order correlations among the four constructs were in the expected directions. Discriminant validity was first examined using the Fornell–Larcker criterion. As shown in Table 4, the square roots of the AVE values for academic major identity (0.749), psychological capital (0.764), career adaptability (0.748), and career decision-making difficulty (0.749) were all greater than the corresponding inter-construct correlations, supporting adequate discriminant validity.

**Table 4:** Correlations and discriminant validity among study variables.

**Variable**	**AMI**	**PsyCap**	**CA**	**CDD**
AMI	0.749			
PsyCap	0.358^***^	0.764		
CA	0.490^***^	0.608^***^	0.748	
CDD	−0.475^***^	−0.482^***^	−0.536^***^	0.749

AMI = academic major identity; PsyCap = psychological capital; CA = career adaptability; CDD = career decision-making difficulty. Values on the diagonal represent the square roots of average variance extracted (AVE). Off-diagonal values represent Pearson correlation coefficients. *p < 0.05, **p < 0.01, ***p < 0.001.

Discriminant validity was further assessed using the heterotrait-monotrait ratio (HTMT). As shown in Table 5, the HTMT values ranged from 0.444 to 0.751, all of which were below commonly recommended cutoff values. These results provide further support for the discriminant validity of the four study constructs.

**Table 5:** Discriminant validity based on the Heterotrait-Monotrait Ratio (HTMT).

**Construct**	**AMI**	**PsyCap**	**CA**	**CDD**
AMI	–			
PsyCap	0.444	–		
CA	0.614	0.751	–	
CDD	0.606	0.609	0.682	–

AMI = academic major identity; PsyCap = psychological capital; CA = career adaptability; CDD = career decision-making difficulty. The values below the diagonal represent the Heterotrait-Monotrait Ratio (HTMT).

### 3.4 Structural model

**Figure 2:**
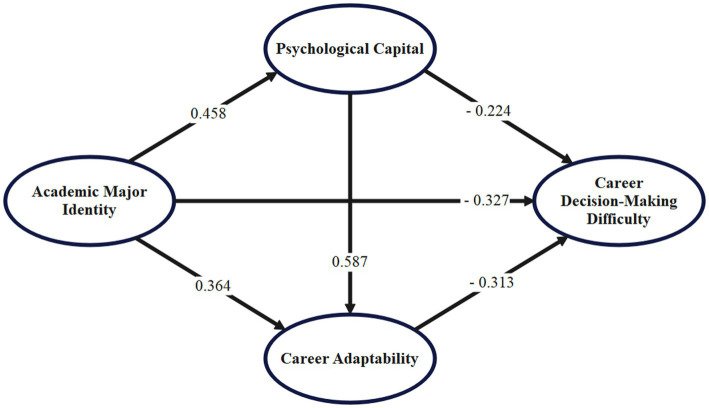
Simplified structural model of the associations among academic major identity, psychological capital, career adaptability, and career decision-making difficulty. Standardized path coefficients are displayed. For readability, only the four higher-order constructs and the main structural paths are shown. All displayed paths were statistically significant at *p* < 0.001. The complete AMOS output, including first-order dimensions and error terms, is provided in Supplementary Figure S1. Detailed structural path estimates are reported in Supplementary Table S2. Arrows indicate hypothesized statistical associations rather than causal effects. AMI = academic major identity; PsyCap = psychological capital; CA = career adaptability; CDD = career decision-making difficulty.

The hypothesized structural model is presented in Figure 2. To improve readability, Figure 2 displays only the four higher-order constructs and the main standardized structural paths. The complete AMOS output, including first-order dimensions and error terms, is provided in Supplementary Figure S1. Detailed structural path estimates corresponding to the paths shown in Figure 2, including standard errors, critical ratios, and *p-*values, are reported in Supplementary Table S2.

The model showed a good fit to the data: χ^2^/df = 3.77, CFI = 0.980, TLI = 0.975, RMSEA = 0.035, and SRMR = 0.031. These fit indices indicate that the proposed pattern of associations among academic major identity, psychological capital, career adaptability, and career decision-making difficulty was consistent with the observed data. Because the structural model was just-identified at the latent level, its fit indices were identical to those of the four-factor measurement model; therefore, interpretation of the structural model focused primarily on the estimated path coefficients.

As shown in Figure 2, all hypothesized direct associations were statistically significant and in the expected direction. Academic major identity was positively associated with psychological capital and career adaptability, and negatively associated with career decision-making difficulty. Psychological capital was positively associated with career adaptability and negatively associated with career decision-making difficulty. Career adaptability was also negatively associated with career decision-making difficulty. Overall, the structural path results were consistent with the hypothesized model.

### 3.5 Bootstrap test of indirect associations

The bias-corrected bootstrap results based on 2,000 bootstrap resamples for the direct, total indirect, specific indirect, sequential indirect, and total associations are presented in Table 6. The direct association between academic major identity and career decision-making difficulty was statistically significant. Bootstrap analyses further showed that the total indirect association and all three specific indirect associations were statistically significant, as none of the 95% bias-corrected confidence intervals included zero.

**Table 6:** Bias-corrected bootstrap results for direct, indirect, sequential indirect, and total associations in the structural model.

Hypothesis	Path	*β*	Boot SE	Boot LLCI	Boot ULCI	Ratio (%)
H1	Direct association: AMI → CDD	−0.327	0.034	−0.394	−0.261	52.07
H2 to H4	Total indirect association	−0.301	0.023	−0.346	−0.255	47.93
H2	AMI → PsyCap → CDD	−0.103	0.020	−0.143	−0.066	16.40
H3	AMI → CA → CDD	−0.114	0.020	−0.152	−0.074	18.15
H4	AMI → PsyCap → CA → CDD	−0.084	0.015	−0.113	−0.054	13.38
	Total association: AMI → CDD	−0.628	0.020	−0.667	−0.591	100

AMI = academic major identity; PsyCap = psychological capital; CA = career adaptability; CDD = career decision-making difficulty. Boot SE = bootstrap standard error; Boot LLCI = lower limit of the 95% bias-corrected confidence interval; Boot ULCI = upper limit of the 95% bias-corrected confidence interval. Ratio (%) represents the proportion of each association relative to the total association. An association was considered statistically significant when the 95% confidence interval did not include zero.

Specifically, academic major identity was indirectly associated with career decision-making difficulty through psychological capital, through career adaptability, and through the sequential pathway of psychological capital and career adaptability. The total association between academic major identity and career decision-making difficulty was also statistically significant. Overall, these findings were consistent with H1, H2, H3, and H4.

## 4 Discussion

The present study examined the associations among academic major identity, psychological capital, career adaptability, and career decision-making difficulty among Chinese college students. Overall, the findings were consistent with the proposed theoretical model and provide an integrated picture of how academic major-related self-perceptions, general positive psychological resources, and career-specific adaptive resources are statistically interconnected with career decision-making difficulty (Zhou W. et al., 2024). Because the study used a cross-sectional design, the findings should be interpreted as associations rather than evidence of causal or temporal processes. Nevertheless, the observed pattern is theoretically meaningful because it links students’ understanding of their academic major with broader psychological resources and career-related adaptive readiness.

### 4.1 Major identity as a basis for career meaning-making

Academic major identity may be understood as more than students’ satisfaction with or preference for a particular field of study. It reflects how students cognitively recognize the value of their major, emotionally accept their academic field, behaviorally engage in major-related learning, and perceive fit between themselves and their major. From a career development perspective, this form of identification may provide an important basis for career meaning-making. Students who develop a stronger sense of identification with their academic major may be more likely to interpret their university learning as coherent, personally meaningful, and connected with possible future careers (Guan et al., 2013; Wang H. et al., 2023).

This interpretation is particularly relevant in the Chinese higher education context. Many students’ major choices are shaped not only by personal interests but also by entrance examination scores, admission adjustment, family expectations, and perceived employment prospects (Guo et al., 2025; Fang et al., 2022). As a result, some students may enter university without a fully stable or internally endorsed sense of fit with their major. Under such circumstances, academic major identity may become an important psychological anchor through which students organize their academic experiences and relate them to future occupational possibilities. Students with higher academic major identity may be more likely to recognize the value of their major, accept their academic role, and interpret major-related experiences in a coherent way. This may correspond to less uncertainty when they consider career-related information, self-understanding, planning, and goals (Lee et al., 2022; Gerçek and Elmas-Atay, 2022).

By contrast, students with lower academic major identity may experience greater hesitation or ambiguity when attempting to translate their academic experiences into career planning. The present findings therefore extend prior research by suggesting that academic major identity is not only relevant to learning engagement, academic adjustment, or career maturity, but also closely linked to students’ career decision-making experiences during the undergraduate years (Liu et al., 2023). From a practical perspective, this highlights the relevance of helping students build a clearer and more positive understanding of their academic majors during the early and middle stages of university life.

### 4.2 Psychological capital and career adaptability as two layers of personal resources

The findings also highlight the importance of distinguishing psychological capital and career adaptability as two related but conceptually different layers of personal resources. Psychological capital reflects relatively general positive psychological resources, including self-efficacy, hope, resilience, and optimism, all of which may shape how students approach academic and career-related tasks (Urbanaviciute et al., 2016; Xu et al., 2023a). A stronger sense of academic major identity may be associated with a more stable and positive interpretation of one’s educational experience. Students who feel cognitively, emotionally, and behaviorally aligned with their academic major may be more likely to perceive themselves as capable of managing academic and future career challenges. They may also be more likely to maintain hope regarding future development, show resilience in the face of uncertainty, and hold a more optimistic outlook. These characteristics are conceptually consistent with higher levels of psychological capital (Elom et al., 2023).

Psychological capital may be especially relevant when students face complex career decisions that require persistence, confidence, self-reflection, information integration, and tolerance of uncertainty. Students with stronger psychological capital may approach career exploration with a greater sense of efficacy and a more constructive orientation, which may correspond to lower levels of career decision-making difficulty. In this sense, psychological capital represents a broader positive psychological correlate linking academic experiences with career-related functioning (Zhou A. et al., 2024; Garcia et al., 2015; Zhou W. et al., 2024). This suggests that the association between academic major identity and career decision-making difficulty is not only academic or contextual in nature, but is also related to broader positive psychological resources (Zhou A. et al., 2024; Liu and Zhang, 2025).

Career adaptability, by contrast, represents a more career-specific set of psychosocial resources for managing career-related tasks and transitions. It includes concern, control, curiosity, and confidence, which are directly connected to students’ engagement with future career issues (Liu et al., 2023). Students who identify more strongly with their academic major may be more likely to maintain future-oriented concern, feel more agentic in career planning, show greater curiosity about occupational possibilities, and approach career tasks with more confidence (Zhang et al., 2021; Rudolph et al., 2017). These adaptive resources may support a more active and organized orientation toward career development.

Career adaptability may also be more proximal to career decision-making difficulty than psychological capital because it is directly tied to the career domain. Students with higher career adaptability may be better able to cope with uncertainty, gather career-relevant information, explore options, and clarify personal goals. These characteristics correspond closely to the types of difficulties reflected in career decision-making difficulty. The present findings therefore suggest that career adaptability may represent a domain-specific career-related correlate of the association between academic major identity and career decision-making difficulty (Leung et al., 2021; Stead et al., 2022). This result extends existing research by showing that academic major identity may be relevant not only to students’ academic functioning, but also to their readiness to engage with career development tasks (Stringer et al., 2011; Zhou A. et al., 2024). In the Chinese context, where university students often face intense pressure to prepare for diverse post-graduation pathways, career adaptability may be especially important (Guan et al., 2013).

Taken together, psychological capital and career adaptability should not be treated as interchangeable constructs. Psychological capital reflects broader positive psychological functioning, whereas career adaptability reflects career-specific adaptive readiness. Considering both constructs in the same model therefore provides a more differentiated explanation of how students’ major-related experiences are associated with career decision-making difficulty.

### 4.3 Interpreting the sequential indirect association from a career construction perspective

The observed sequential indirect association is consistent with the theoretical ordering proposed in the present model, but it should not be interpreted as evidence of a confirmed developmental sequence. From a career construction perspective, students respond to career-related tasks and transitions by constructing meaning and drawing on psychosocial resources (Savickas, 2005). In this framework, academic major identity may be viewed as a form of major-related meaning construction, psychological capital as a general layer of positive psychological resources, and career adaptability as a more career-specific layer of adaptive resources.

This interpretation helps explain why psychological capital and career adaptability may be statistically linked within the broader association between academic major identity and career decision-making difficulty. Psychological capital captures relatively general positive psychological capacities, whereas career adaptability reflects more domain-specific psychosocial resources for dealing with career-related tasks and transitions (Liu and Zhang, 2025; Zhou B. et al., 2024; Xu M. et al., 2023). These two constructs may therefore represent different but related levels of personal resources. Broader positive psychological resources may be statistically linked to career-specific adaptive readiness, especially when students are facing complex career decision-making tasks (Liu and Zhang, 2025).

From this perspective, academic major identity may be associated with how students understand and experience their university education, and this may correspond to stronger positive psychological resources (Zhou A. et al., 2024). These positive resources may then be linked to higher career adaptability, which in turn may correspond to lower career decision-making difficulty. Although these data do not permit causal inference, the observed statistical pattern is consistent with the theoretical ordering and provides a useful integrative framework for understanding the interrelations among academic, psychological, and career-related variables in Chinese college students (Zhou W. et al., 2024; Fang et al., 2025).

Importantly, the sequential indirect association suggests that academic major identity may be connected to career decision-making difficulty through multiple, interrelated pathways rather than through a single direct link alone (Zhuang et al., 2018). This multidimensional pattern may help explain why some students with stronger major identification also report more positive psychological functioning and greater readiness to manage career development tasks (Jiang et al., 2022). Nevertheless, alternative directions are also plausible. For example, students with less career decision-making difficulty may develop more positive views of their major, and students with stronger career adaptability may report higher psychological capital. Therefore, the sequential pattern should be understood as a theoretically informed statistical model rather than a confirmed causal process.

### 4.4 Theoretical and practical implications

The present findings offer several theoretical implications. First, by showing that academic major identity, psychological capital, career adaptability, and career decision-making difficulty were systematically related at the construct level, this study connects lines of research that are often examined separately, namely academic-major-related experiences, positive psychological resources, and career-related adaptive functioning (Zhou W. et al., 2024; Liu and Zhang, 2025). Rather than treating career decision-making difficulty as an isolated outcome, the findings place it within a broader pattern of associations involving both major-related identification and personal resources (Zhou A. et al., 2024; Jia et al., 2022).

Second, the results clarify the different roles of psychological capital and career adaptability. Psychological capital reflects relatively general positive psychological resources, whereas career adaptability is more specifically tied to career-related readiness and coping. The fact that both constructs showed indirect associations, including a sequential indirect pattern, supports the value of considering both broader psychological functioning and more domain-specific adaptive resources when examining career decision-making difficulty among college students (Xu et al., 2023a; Jia et al., 2022).

Third, the study contributes to research on career development in the Chinese higher education context. In China, academic major experiences are often closely tied to institutional placement, family expectations, and anticipated employment prospects (Tian and Chen, 2020; Guan et al., 2015; Yang et al., 2024). Against this background, academic major identity may be particularly relevant because students need to make sense of their university experiences while preparing for increasingly diverse career possibilities. Although the current data do not speak to causal ordering, they support the usefulness of examining these constructs within an integrated framework in Chinese higher education settings (Liu et al., 2023; Fang et al., 2025).

The practical implications of the present study should be interpreted with appropriate caution. Given the cross-sectional design, the findings do not justify claims about intervention effects or directional influence. Even so, the observed associations may still be informative for student support in universities. First, universities may benefit from paying attention to whether students have sufficient opportunities to understand the content, value, and possible career relevance of their majors, especially during the early and middle years of undergraduate study (Xia et al., 2025; Krieshok et al., 2009). Major orientation, academic advising, curriculum-based career education, and opportunities to learn about major-related occupations may help students connect academic learning with future pathways.

Second, because psychological capital and career adaptability were both linked to career decision-making difficulty, student support services may consider these constructs as potentially useful assessment or counseling reference points (Luthans and Youssef-Morgan, 2017). For example, when students report substantial difficulty in career decision-making, it may be helpful to also consider whether they show relatively low levels of confidence, hope, resilience, optimism, future concern, control, curiosity, or confidence in the career domain. This does not mean that improving these factors would necessarily reduce career decision-making difficulty, but it does suggest that these dimensions may be relevant in understanding students’ career-related experiences (Zhou W. et al., 2024; Leung et al., 2021).

Third, the observed pattern suggests that academic advising, psychological support, and career services may be better viewed as complementary rather than fully separate domains. Academic experiences, general psychological resources, and career-related adaptive resources may be intertwined in students’ reports (Song et al., 2024; Xiong et al., 2026). For universities in China, this may be especially relevant when designing integrated student support systems for undergraduates facing increasingly diverse post-graduation options.

### 4.5 Limitations and future directions

Several limitations should be noted. First, the study used a cross-sectional design. Therefore, the associations identified in this study should not be interpreted as evidence of temporal ordering or causal processes (Maxwell and Cole, 2007). This point is particularly important for the sequential indirect pattern involving psychological capital and career adaptability. Although the statistical model was consistent with the hypothesized ordering, alternative orderings and reciprocal relations are also possible. Future research could use longitudinal, cross-lagged, or multi-wave designs to examine whether similar patterns emerge across time and to better evaluate temporal relations among the constructs (Taris and Kompier, 2003).

Second, the sample was drawn from 20 universities in Sichuan Province and included only first-, second-, and third-year undergraduates. This sampling strategy was appropriate for the present study, but it also limits the scope of generalization. The pattern of associations observed here may differ in other regions of China because of differences in economic development, higher education resources, local labor markets, and cultural expectations regarding education and employment. The use of convenience sampling and voluntary participation may also have introduced selection bias, as students who were more interested in career development or more willing to complete an online questionnaire may have been more likely to participate. In addition, senior students were not included, even though they may face more immediate graduation-related pressures. Future studies could test whether the present findings replicate in broader national samples and could further compare different grade groups, especially by including senior students or examining transition-stage samples separately (Hsieh, 2025).

Third, the sample structure should be considered when interpreting the findings. The final sample included a higher proportion of students from urban areas than from rural areas, and only a minority of participants reported internship experience. These characteristics may be relevant because urban and rural students may differ in access to career information, family resources, and employment expectations, whereas students with internship experience may have different levels of career clarity and occupational information. Although the sample covered multiple academic disciplines, the analyses were conducted at the overall sample level. It therefore remains unclear whether the strength of the associations differs across disciplinary contexts such as humanities and social sciences, science and engineering, medicine, or arts and sports (Holland, 1997; Diniz et al., 2025). Given that academic major identity may have different meanings across fields, future research could examine subgroup differences or test measurement invariance and structural invariance across disciplinary categories (Putnick and Bornstein, 2016).

Fourth, all variables were assessed through self-report questionnaires administered in a single online session. Although procedural remedies and CFA-based diagnostic analyses were used, common method variance cannot be fully ruled out (Podsakoff et al., 2003). In particular, no theoretically unrelated marker variable was included, so a marker-variable approach could not be implemented in the present study. Moreover, the association between psychological capital and career adaptability should be interpreted cautiously because both constructs were measured by self-report and both capture positive resource-related perceptions. Future research could incorporate marker variables, multiple informants, behavioral indicators, or mixed-method designs to reduce reliance on a single method.

Fifth, all measures were administered using a unified 5-point Likert format. This approach helped reduce response burden and cognitive switching in the online survey, but it also meant that the original response formats of some instruments were adapted (Friborg et al., 2006). Although reliability, convergent validity, and discriminant validity were examined in the present sample, future studies could test whether the observed pattern of associations remains robust when the original response formats of the instruments are retained.

Sixth, the present analyses focused primarily on higher-order construct scores. This approach was appropriate for testing the overall conceptual model, but it may have obscured potentially informative differences among first-order dimensions (Schneider et al., 1996). For example, different aspects of academic major identity or career adaptability may not relate to career decision-making difficulty in exactly the same way (Rudolph et al., 2017). Some first-order dimensions of career decision-making difficulty also showed comparatively lower reliability than the corresponding higher-order construct, although they remained within acceptable ranges. Future research could conduct dimension-level analyses or sensitivity analyses to examine whether the findings are robust across different measurement specifications.

Finally, the present study focused on four constructs that were theoretically relevant to the proposed model, but career decision-making difficulty is likely to be related to additional contextual and developmental factors, such as family expectations, perceived labor market uncertainty, career decision-making self-efficacy, social support, and prior career exploration experiences. Incorporating these factors into future models may help provide a more differentiated understanding of career decision-making difficulty among Chinese college students (Leung et al., 2011; Zhu et al., 2023).

## 5 Conclusion

In a sample of Chinese college students, academic major identity was negatively associated with career decision-making difficulty, and this association was also reflected in indirect links involving psychological capital and career adaptability, including a sequential indirect pattern. Overall, the findings support an integrated view in which academic major identity, general positive psychological resources, career-related adaptive resources, and career decision-making difficulty are meaningfully interconnected in the Chinese undergraduate context.

## Data Availability

The original contributions presented in the study are included in the article/Supplementary material, further inquiries can be directed to the corresponding author/s.
