# Supplementary material for: The association between academic major identity and career decision-making difficulty among Chinese college students: a sequential indirect association model of psychological capital and career adaptability
Source: Front Psychol. 2026 Jun 17;17:1869688. doi: 10.3389/fpsyg.2026.1869688 (PMC13319036; doi:10.3389/fpsyg.2026.1869688)
Supplement: Supplementary file 1 [file Supplementary_file_1.docx]

## Supplementary Table S1. Measurement model results for the first-order dimensions and higher-order reflective constructs.

| **Construct** | **Dimension / Subdimension** | **No. of items** | **Cronbach’s α** | **Std. Loading range** | **CR** | **AVE** |
| --- | --- | --- | --- | --- | --- | --- |
| Academic Major Identity | Cognitive Identity | 5 | 0.882 | 0.754-0.842 | 0.884 | 0.604 |
|  | Affective Identity | 8 | 0.906 | 0.716-0.761 | 0.907 | 0.548 |
|  | Behavioral Identity | 6 | 0.863 | 0.657-0.753 | 0.865 | 0.517 |
|  | Fit | 4 | 0.831 | 0.734-0.777 | 0.839 | 0.566 |
|  | **Higher-order construct** | **23** | **0.929** | **0.723-0.768** | **0.836** | **0.561** |
| Psychological Capital | Self-Efficacy | 6 | 0.904 | 0.673-0.865 | 0.906 | 0.617 |
|  | Hope | 6 | 0.891 | 0.707-0.856 | 0.893 | 0.584 |
|  | Resilience | 6 | 0.883 | 0.686-0.777 | 0.885 | 0.562 |
|  | Optimism | 6 | 0.891 | 0.709-0.789 | 0.891 | 0.578 |
|  | **Higher-order construct** | **24** | **0.940** | **0.739-0.778** | **0.849** | **0.584** |
| Career Adaptability | Concern | 6 | 0.902 | 0.714-0.805 | 0.903 | 0.608 |
|  | Control | 6 | 0.905 | 0.752-0.849 | 0.907 | 0.620 |
|  | Curiosity | 6 | 0.891 | 0.753-0.765 | 0.891 | 0.578 |
|  | Confidence | 6 | 0.874 | 0.576-0.824 | 0.879 | 0.550 |
|  | **Higher-order construct** | **24** | **0.937** | **0.730-0.763** | **0.836** | **0.560** |
| Career Decision-Making Difficulty | Difficulties in Career Information Exploration | 5 | 0.881 | 0.735-0.826 | 0.882 | 0.600 |
|  | Difficulties in Career Self-Exploration | 4 | 0.814 | 0.695-0.768 | 0.817 | 0.528 |
|  | Difficulties in Career Planning Exploration | 3 | 0.783 | 0.678-0.784 | 0.784 | 0.548 |
|  | Difficulties in Career Goal Exploration | 4 | 0.800 | 0.655-0.744 | 0.806 | 0.510 |
|  | **Higher-order construct** | **16** | **0.898** | **0.736-0.764** | **0.836** | **0.561** |

## **Note.** Std. loading range = standardized factor loading range; CR = composite reliability; AVE = average variance extracted. All Cronbach’s α and CR values exceeded 0.70, and all AVE values exceeded 0.50. Although several first-order dimensions of career decision-making difficulty showed comparatively lower reliability than the corresponding higher-order construct, their reliability indices remained within acceptable ranges. Therefore, the main structural analyses were interpreted primarily at the higher-order construct level.

##
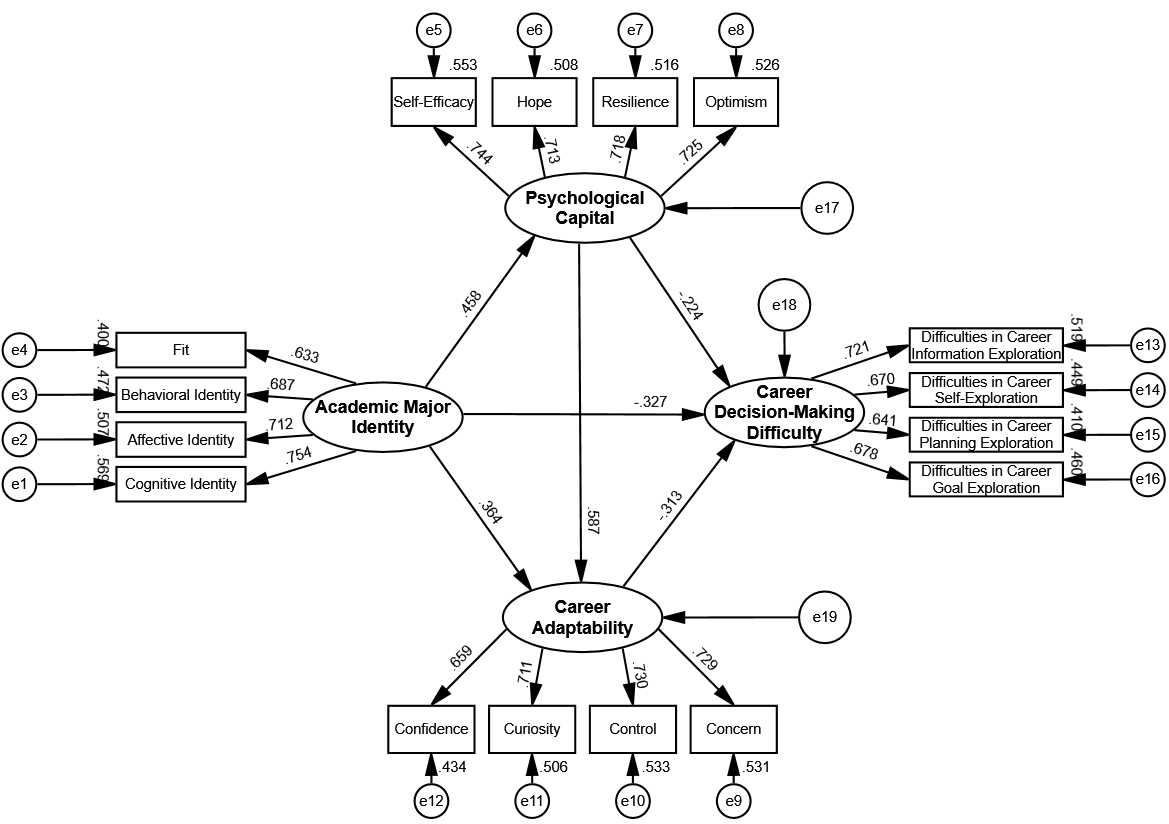
Supplementary Figure S1. Complete measurement and structural model estimated in AMOS.

## Note. The figure shows the complete AMOS-estimated model, including the four higher-order constructs, their first-order dimensions, measurement residuals, and standardized structural paths. AMI = academic major identity; PsyCap = psychological capital; CA = career adaptability; CDD = career decision-making difficulty. Standardized coefficients are displayed. To improve readability, the main text presents a simplified structural model showing only the higher-order constructs and the principal structural paths in Figure 2. Arrows represent hypothesized statistical associations rather than causal effects.

## Supplementary Table S2. Standardized structural path estimates for the hypothesized structural model.

| **Path** | **Standardized coefficient (β)** | **SE** | **C.R.** | **p** |
| --- | --- | --- | --- | --- |
| AMI→PsyCap | 0.458 | 0.028 | 16.938 | < 0.001 |
| AMI→CA | 0.364 | 0.025 | 14.336 | < 0.001 |
| AMI→CDD | -0.327 | 0.030 | -10.194 | < 0.001 |
| PsyCap→CA | 0.587 | 0.027 | 20.803 | < 0.001 |
| PsyCap→CDD | -0.224 | 0.037 | -5.644 | < 0.001 |
| CA→CDD | -0.313 | 0.047 | -6.375 | < 0.001 |

**Note.** AMI = academic major identity; PsyCap = psychological capital; CA = career adaptability; CDD = career decision-making difficulty. β = standardized coefficient; SE = standard error; C.R. = critical ratio. All paths were statistically significant at p < .001. Arrows indicate hypothesized statistical associations rather than causal effects.
